# Correction: Enhanced desalination with polyamide thin-film membranes using ensemble ML chemometric methods and SHAP analysis

**DOI:** 10.1039/d4ra90141j

**Published:** 2024-11-28

**Authors:** Jamilu Usman, Sani I. Abba, Fahad Jibrin Abdu, Lukka Thuyavan Yogarathinam, Abdullah G. Usman, Dahiru Lawal, Billel Salhi, Isam H. Aljundi

**Affiliations:** a Interdisciplinary Research Centre for Membranes and Water Security (IRC-MWS), King Fahd University of Petroleum and Minerals Dhahran 31261 Saudi Arabia saniisaabba86@gmail.com; b Department of Chemical Engineering, Prince Mohammad Bin Fahd University Al Khobar 31952 Saudi Arabia; c Water Research Centre, Prince Mohammad Bin Fahd University Al Khobar 31952 Saudi Arabia; d SADAIA-KFUPM Joint Research Center for Artificial Intelligence (JRCAI), King Fahd University of Petroleum & Minerals (KFUPM) Dhahran Saudi Arabia; e Near East University, Operational Research Center in Healthcare Nicosia, TRNC 10 Mersin 99138 Turkey; f Mechanical Engineering Department, King Fahd University of Petroleum & Minerals Dhahran 31261 Saudi Arabia; g Chemical Engineering Department, King Fahd University of Petroleum and Minerals Dhahran 31261 Saudi Arabia

## Abstract

Correction for ‘Enhanced desalination with polyamide thin-film membranes using ensemble ML chemometric methods and SHAP analysis’ by Jamilu Usman *et al.*, *RSC Adv.*, 2024, **14**, 31259–31273, https://doi.org/10.1039/D4RA06078D.

The authors regret the omission of an acknowledgements section in the original article. The acknowledgements are given below.

This publication is based upon work supported by King Fahd University of Petroleum & Minerals. The author(s) at KFUPM acknowledge the Interdisciplinary Research Center for Membranes & Water Security and DROC for the support received. This research was funded by the Deanship of Research Oversight and Coordination (DROC) at King Fahd University of Petroleum & Minerals (KFUPM) under the Interdisciplinary Research Center for Membranes and Water Security.

The Royal Society of Chemistry apologises for these errors and any consequent inconvenience to authors and readers.

